# Effect of Pemafibrate on Metabolic Dysfunction‐Associated Steatotic Liver Disease: A Nationwide Multicenter Study

**DOI:** 10.1002/jgh3.70277

**Published:** 2025-09-09

**Authors:** Yasuyuki Komiyama, Nobuharu Tamaki, Keiji Tsuji, Nami Mori, Toshie Mashiba, Hironori Ochi, Haruhiko Kobashi, Chikara Ogawa, Michiko Nonogi, Hideo Yoshida, Takehiro Akahane, Masahiko Kondo, Toyotaka Kasai, Hideki Fujii, Yasushi Uchida, Hirotaka Arai, Kaoru Tsuchiya, Namiki Izumi, Masayuki Kurosaki

**Affiliations:** ^1^ Department of Gastroenterology and Hepatology Musashino Red Cross Hospital Tokyo Japan; ^2^ Department of Gastroenterology Hiroshima Red Cross Hospital and Atomic‐Bomb Survivors Hospital Hiroshima Japan; ^3^ Center for Liver‐Biliary‐Pancreatic Disease, Matsuyama Red Cross Hospital Matsuyama Japan; ^4^ Department of Gastroenterology Japanese Red Cross Okayama Hospital Okayama Japan; ^5^ Department of Gastroenterology and Hepatology Takamatsu Red Cross Hospital Takamatsu Kagawa Japan; ^6^ Department of Gastroenterology Tokushima Red Cross Hospital Tokushima Japan; ^7^ Department of Gastroenterology Japanese Red Cross Medical Center Tokyo Japan; ^8^ Department of Gastroenterology Ishinomaki Red Cross Hospital Ishinomaki Japan; ^9^ Department of Gastroenterology Otsu Red Cross Hospital Otsu Japan; ^10^ Department of Gastroenterology Fukaya Red Cross Hospital Saitama Japan; ^11^ Department of Gastroenterology Japanese Red Cross Kyoto Daiichi Hospital Kyoto Japan; ^12^ Department of Gastroenterology Matsue Red Cross Hospital Matsue Japan; ^13^ Department of Gastroenterology Maebashi Red Cross Hospital Maebashi Japan

**Keywords:** hypertriglyceridemia, liver function, metabolic dysfunction‐associated steatotic liver disease, multicenter study, pemafibrate

## Abstract

**Aim:**

Although pemafibrate, a selective peroxisome proliferator‐activated receptor α (PPARα) modulator primarily prescribed for hypertriglyceridemia, may improve liver function in patients with metabolic dysfunction‐associated steatotic liver disease (MASLD), its efficacy has not been sufficiently evaluated. This study aimed to assess the effect of pemafibrate on liver function in patients with MASLD using a nationwide multicenter cohort.

**Methods:**

In this nationwide multicenter study, we analyzed 352 patients diagnosed with MASLD and hypertriglyceridemia who newly initiated pemafibrate therapy. The primary outcome was the rate of ALT normalization (ALT ≤ 30 U/L). Laboratory data were collected at baseline and 3, 6, and 12 months after pemafibrate initiation, and longitudinal changes were evaluated.

**Results:**

The mean ALT levels decreased significantly from 60.1 ± 1.8 U/L at baseline to 44.9 ± 1.4, 41.6 ± 1.3, and 38.1 ± 1.3 U/L at 3, 6, and 12 months, respectively (all *p* < 0.001). The ALT normalization rate increased from 30.4% at baseline to 46.0%, 50.9%, and 56.6% at 3, 6, and 12 months (*p* < 0.001), respectively, with marked improvement at three months and maintenance throughout follow‐up.

**Conclusions:**

Pemafibrate treatment significantly improved ALT levels and ALT normalization rates in MASLD patients with hypertriglyceridemia, with improvement observed at three months and a sustained effect up to 12 months. This is a large‐scale multicenter study to date evaluating pemafibrate in MASLD, providing robust evidence for its potential as a therapeutic option in this population.

**Abbreviations**
Alb: albumin
ALT: alanine aminotransferase
AST: aspartate aminotransferase
BMI: body mass index
GGT: gamma‐glutamyl transferase
HDL‐C: high‐density lipoprotein cholesterol
IQR: interquartile range
LDL‐C: low‐density lipoprotein cholesterol
MASLD: metabolic dysfunction‐associated steatotic liver disease
NAFLD: nonalcoholic fatty liver disease
SE: standard error
T‐Bil: total bilirubin
TG: triglycerides

## 1 Introduction

Metabolic dysfunction‐associated steatotic liver disease (MASLD) has recently undergone a global redefinition and is drawing increasing attention in the field of hepatology [1, 2, 3, 4]. MASLD, which encompasses the former concept of nonalcoholic fatty liver disease (NAFLD) [5, 6, 7], is associated not only with the pathogenesis of hepatocellular carcinoma and hepatic fibrosis progression but also with an increased risk of cardiovascular events and extrahepatic malignancies, making it a medical condition of significant social and clinical importance [8, 9, 10, 11, 12].

While preventing MASLD‐induced cirrhosis and liver cancer is an important treatment goal, there is still a paucity of effective pharmacotherapies for MASLD. However, many patients with MASLD have comorbidities such as diabetes mellitus and dyslipidemia, and some drugs used to treat these conditions may also have beneficial effects on MASLD itself. Pemafibrate, a selective peroxisome proliferator‐activated receptor α (PPARα) modulator, is mainly prescribed for hypertriglyceridemia [13]. Recent multicenter studies have reported that pemafibrate improves liver biochemistry and prognostic scores in patients with primary biliary cholangitis [14]. Several reports have suggested that this medication may improve the levels of ALT and noninvasive fibrosis markers in MASLD [15, 16, 17, 18, 19, 20, 21, 22, 23, 24, 25, 26, 27]; however, these studies have generally been single‐center or relatively small in scale, with limited sample sizes. To date, the effect of pemafibrate on ALT improvement in patients with MASLD has not been sufficiently evaluated in large, multicenter cohort studies.

Therefore, to fill this knowledge gap, we conducted a nationwide multicenter study including 13 Japanese Red Cross hospitals, which comprise hospitals distributed throughout Japan and facilitate robust multicenter data collection, to evaluate the impact of pemafibrate therapy on longitudinal changes in ALT levels and the rate of ALT normalization in patients with MASLD.

## 2 Materials and Methods

### 2.1 Study Protocol

This was a nationwide, multicenter, retrospective cohort study conducted by the Japanese Red Cross Liver Study Group. Patients diagnosed with MASLD and hypertriglyceridemia who had newly initiated pemafibrate therapy (0.2 mg/day) between 2019 and 2023 were included. In this study, fatty liver was diagnosed based on evidence of hepatic steatosis on abdominal ultrasonography, computed tomography, and magnetic resonance imaging (MRI).

Patients with active viral hepatitis (Patients who were HBsAg‐positive or HCV RNA‐positive were excluded, whereas those with SVR more than 3 years after HCV treatment were included), autoimmune liver diseases, primary biliary cholangitis, drug‐induced liver injury, or alcoholic liver disease were excluded from the analysis.

Informed consent was obtained from all patients using the opt‐out method. This retrospective cohort study was approved by the ethics review committees of Musashino Red Cross Hospital (approval number: 4072) and conformed to the ethical guidelines of the Declaration of Helsinki.

### 2.2 Clinical and Laboratory Data

Clinical background data (including age, sex, comorbidities, body mass index [BMI], and the presence of diabetes mellitus, hypertension, or dyslipidemia) and laboratory values at baseline (pretreatment) and 3, 6, and 12 months after initiation of therapy were collected. The laboratory parameters analyzed included serum levels of alanine aminotransferase (ALT), gamma‐glutamyl transferase (GGT), and triglycerides (TG).

### 2.3 Primary Outcome

This study's primary outcome was the rate of ALT normalization. ALT values were assessed at baseline and 3, 6, and 12 months after pemafibrate initiation. ALT normalization was defined as an ALT level of ≤ 30 U/L.

### 2.4 Statistical Analyses

Continuous variables are presented as the mean ± standard error (SE) for normally distributed data and median values with interquartile ranges for non‐normally distributed data. Comparisons between the baseline and each time point (3, 6, and 12 months) were performed using paired *t*‐tests or the Mann–Whitney U test for normally distributed and non‐normally distributed data, respectively. For changes in the rate of ALT normalization over time, Cochran's Q test was used to assess overall significance.

A two‐sided *p*‐value of < 0.05 was considered statistically significant. All statistical analyses were performed using EZR (Saitama Medical Center, Jichi Medical University, Saitama, Japan), which is a graphical user interface for R (The R Foundation for Statistical Computing, Vienna, Austria) [28].

We additionally performed a multivariable logistic regression to identify predictors of ALT normalization at 12 months. Triglycerides were categorized as < 200, 200–299, 300–399, and ≥ 400 mg/dL; covariates included age, sex, BMI, baseline ALT, and platelet count.

## 3 Results

### 3.1 Patient Characteristics

A total of 352 patients were included in this study, and their baseline characteristics are summarized in Table 1. The median age was 56 years (IQR, 48–67), and 220 patients (62.5%) were male. The median BMI was 27.1 (24.4–30.1) kg/m^2^. The prevalence of diabetes mellitus and hypertension was 43.4% and 61.2%, respectively. The median (IQR) values for key laboratory parameters at baseline were as follows: albumin, 4.4 (4.1–4.6) g/dL; total bilirubin, 0.7 (0.5–0.8) mg/dL; AST, 35 (24–57) U/L; ALT, 45 (25–81) U/L; GGT, 65 (35–128) U/L; and triglycerides, 300 (192–472) mg/dL.

**TABLE 1:** Baseline characteristics of the study participants.

	*n* = 352
Age, years	56 (48–67)
Male, %	220 (62.5)
BMI, kg/m^2^	27.1 (24.4–30.1)
Diabetes, %	126 (43.4)
Hypertension, %	194 (61.2)
Alb, g/dL	4.4 (4.1–4.6)
T–Bli, mg/dL	0.7 (0.5–0.8)
AST, U/L	35 (24–57)
ALT, U/L	45 (25–81)
GGT, U/L	65 (35–128)
LDL–C, mg/dL	114 (89–140)
HDL–C, mg/dL	45 (3855)
TG, mg/dL	300 (192–472)
Platelet, 10^9^/L	228 (186–271)

*Note:* Data are presented as the median (interquartile range) or *n* (%).

Abbreviations: Alb, albumin; ALT, alanine aminotransferase; AST, aspartate aminotransferase; BMI, body mass index; GGT, gamma‐glutamyl transferase; HDL‐C, high‐density lipoprotein cholesterol; LDL‐C, low‐density lipoprotein cholesterol; T‐Bil, total bilirubin; TG, triglycerides.

### 3.2 Changes in Biochemical Parameters During Follow‐Up

**FIGURE 1:**
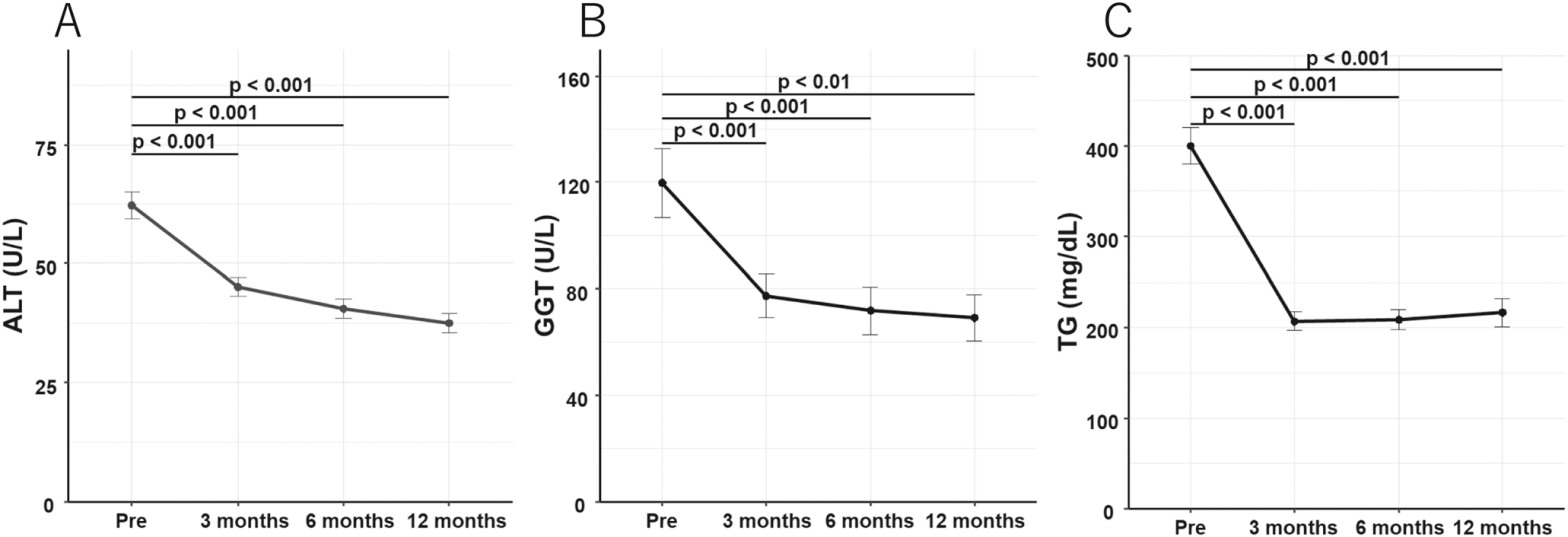
Changes in ALT, GGT, and TG levels during pemafibrate therapy. (A) ALT, (B) GGT, and (C) TG. Mean values (± standard error) at baseline (Pre) and at 3, 6, and 12 months after initiation of pemafibrate are shown. Paired *t*‐tests were performed to compare the baseline with each time point. Horizontal lines and *p*‐values above the plots indicate statistically significant differences between the baseline and each follow‐up time point (paired *t*‐test).

The longitudinal changes in biochemical parameters are shown in Figure 1. (A) The mean ALT level was 60.1 ± 1.8 U/L at baseline, which significantly decreased to 44.9 ± 1.4 U/L at 3 months, 41.6 ± 1.3 U/L at 6 months, and 38.1 ± 1.3 U/L at 12 months (all *p* < 0.001 vs. baseline). The mean changes in ALT from baseline were –16.8 ± 38.0 U/L at 3 months, −17.8 ± 34.7 U/L at 6 months, and –18.5 ± 37.2 U/L at 12 months. (B) Mean GGT levels also decreased significantly from 119.9 ± 12.8 U/L at baseline to 77.5 ± 8.2 U/L at 3 months, 71.9 ± 8.9 U/L at 6 months, and 69.1 ± 8.6 U/L at 12 months (all *p* < 0.01 vs. baseline). (C) The mean TG value decreased from 400.2 ± 19.9 mg/dL at baseline to 206.9 ± 10.3 mg/dL at 3 months, 208.4 ± 11.0 mg/dL at 6 months, and 216.3 ± 15.5 mg/dL at 12 months (all *p* < 0.001 vs. baseline).

### 3.3 ALT Normalization Rate in Patients Treated With Pemafibrate

**FIGURE 2:**
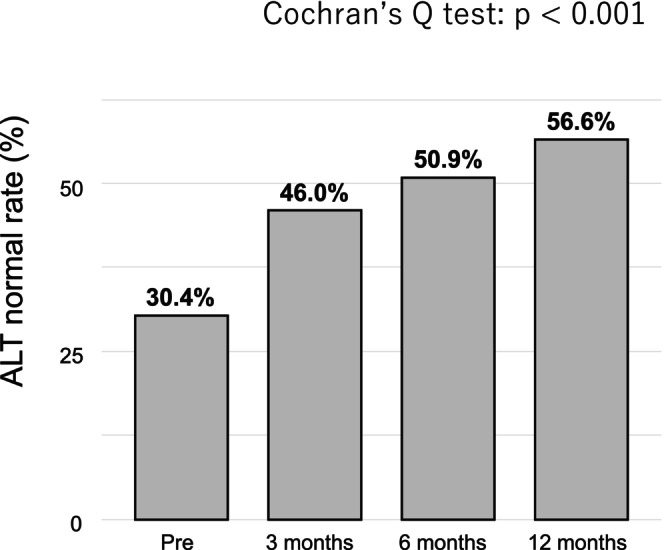
Changes in the proportion of patients with normal ALT levels during pemafibrate therapy. The bar graph shows the proportion of patients with normal ALT (ALT ≤ 30 U/L) values at baseline (Pre) and at 3, 6, and 12 months after the initiation of pemafibrate therapy. The ALT normalization rate increased significantly over time (Cochran's Q test, *p* < 0.001).

The temporal changes in the proportion of patients with normal ALT levels are shown in Figure 2. The ALT normalization rate significantly increased from 30.4% at baseline to 46.0% at 3 months, 50.9% at 6 months, and 56.6% at 12 months (Cochran's Q test, *p* < 0.001). Notably, a marked improvement was observed at three months, and this effect was sustained throughout the treatment period. Both triglyceride and ALT levels improved at 3 months and were maintained up to 12 months, with the proportion of ALT normalization gradually increasing during the follow‐up period. In the adjusted model, triglycerides ≥ 400 mg/dL were independently associated with ALT normalization compared with < 200 mg/dL (adjusted OR 3.30; 95% CI, 1.01–10.8; *p* = 0.048), while age and baseline ALT were also significant; other covariates were not (Table 2).

**TABLE 2:** Multivariable logistic regression for predictors of ALT normalization at 12 months.

Variable	Adjusted OR	95% CI	*p*
*TG*			
< 200 mg/dL	1.00	Reference	—
200–299 mg/dL	0.92	0.32–2.69	0.883
300–399 mg/dL	1.10	0.31–3.93	0.885
≥ 400 mg/dL	3.30	1.01–10.8	0.048
Age (per 1 year)	1.04	1.00–1.08	0.033
Male sex	1.58	0.61–4.07	0.349
BMI (per 1 kg/m2)	0.97	0.89–1.05	0.447
ALT (per 10 U/L)	0.68	0.57–0.79	< 0.001
Platelet (per 10 × 10^9^/L)	1.22	0.67–2.16	0.585

*Note:* Data are presented as adjusted odds ratios (ORs) with 95% confidence intervals (CIs). Triglycerides < 200 mg/dL were used as the reference category. ALT was modeled per 10 U/L, and platelet count per 10 × 10^9^/L increase.

Abbreviations: ALT, alanine aminotransferase; BMI, body mass index; CI, confidence interval; OR, odds ratio; TG, triglycerides.

## 4 Discussion

### 4.1 Main Findings

This study demonstrated that pemafibrate therapy led to sustained improvements in ALT levels and increased rates of ALT normalization in patients with MASLD. Importantly, this represents a large‐scale multicenter investigation of pemafibrate in MASLD to date, providing more robust evidence than previous smaller studies.

### 4.2 In the Context of Published Literature

In MASLD, hepatic fibrosis is most strongly associated with prognosis, and an improvement in fibrosis is considered the key therapeutic target. However, repeated liver biopsies are required to assess fibrosis, which is unfeasible in routine clinical practice. Although ALT is a marker of liver injury, a longitudinal improvement in ALT levels correlates with improvements in fibrosis and inflammation per previous reports, making it a practical short‐term indicator of treatment efficacy [29, 30, 31, 32, 33]. Therefore, ALT normalization serves as a clinically meaningful surrogate endpoint. Diet and exercise are the cornerstone of MASLD treatment, and structured lifestyle programs have been shown to improve liver function tests and body weight [34]. However, these interventions often fail, indicating that pharmacological treatment is also desirable. Early identification and treatment of high‐risk MASLD patients is essential, as delayed intervention may worsen hepatic and extrahepatic outcomes [35]. In the present study, we demonstrated that pemafibrate administration in MASLD patients with hypertriglyceridemia led to significant improvements and the normalization of ALT levels. Notably, this effect appeared as early as 3 months and was sustained through 12 months. While previous studies have reported the beneficial effects of pemafibrate on MASLD, these were limited to single‐center or small‐scale investigations to the best of our knowledge [15, 16, 17, 18, 19, 20, 21, 22, 23, 24, 25, 26, 27]. The present multicenter cohort provides more robust evidence supporting the efficacy of pemafibrate for MASLD. As the development of effective pharmacotherapies for MASLD remains an unmet need, our findings suggest that pemafibrate could be a promising treatment option for this condition.

### 4.3 Strengths and Limitations

The main strength of this study is its large‐scale cohort design using a nationwide, multicenter database. However, several limitations should be noted. First, this was a retrospective study, which may be subject to unmeasured confounders and selection bias. Second, liver biopsy was not performed at baseline or during follow‐up; therefore, histological diagnoses of MASH and longitudinal assessment of fibrosis were not available. Third, liver‐related outcomes other than ALT levels, as well as broader clinical endpoints, could not be fully evaluated. Finally, the observation period was limited to 12 months, which may not capture long‐term effects.

### 4.4 Future Implications

The development of effective pharmacotherapies for MASLD remains an important unmet clinical need. While statins are recommended in current guidelines for the management of dyslipidemia in MASLD, fibrates are not currently endorsed [29, 30, 31, 32]. Our findings suggest that pemafibrate may improve ALT levels and could be considered a promising therapeutic option for patients with MASLD. In addition to triglyceride lowering, pemafibrate has been reported to promote hepatic β‐oxidation, improve lipid transport and mitochondrial function, and exert anti‐inflammatory and antioxidant effects, which may collectively contribute to improvements in liver function [16]. Further studies are warranted to determine whether pemafibrate can also improve hepatic fibrosis and long‐term outcomes—such as the prevention of cirrhosis and hepatocellular carcinoma—in MASLD.

In conclusion, pemafibrate treatment resulted in a significant reduction in ALT levels and an increased rate of ALT normalization in patients with MASLD and hypertriglyceridemia.

## Conflicts of Interest

Nobuharu Tamaki received a lecture fee from Kowa Company. The other authors declare no conflicts of interest.

## Data Availability

The data that support the findings of this study are available on request from the corresponding author. The data are not publicly available due to privacy or ethical restrictions.

## References

[1] R. Loomba and A. J. Sanyal , “The Global NAFLD Epidemic,” Nature Reviews. Gastroenterology and Hepatology 10, no. 11 (2013): 686–690. 24042449 10.1038/nrgastro.2013.171

[2] L. Y. Mak , K. Liu , S. Chirapongsathorn , et al., “Liver Diseases and Hepatocellular Carcinoma in the Asia‐Pacific Region: Burden, Trends, Challenges and Future Directions,” Nature Reviews. Gastroenterology and Hepatology 21, no. 12 (2024): 834–851. 39147893 10.1038/s41575-024-00967-4

[3] H. Fujii , Y. Suzuki , K. Sawada , et al., “Prevalence and Associated Metabolic Factors of Nonalcoholic Fatty Liver Disease in the General Population From 2014 to 2018 in Japan: A Large‐Scale Multicenter Retrospective Study,” Hepatology Research 53, no. 11 (2023): 1059–1072. 37537735 10.1111/hepr.13947

[4] H. Enomoto , N. Akuta , H. Hikita , et al., “Etiological Changes of Liver Cirrhosis and Hepatocellular Carcinoma‐Complicated Liver Cirrhosis in Japan: Updated Nationwide Survey From 2018 to 2021,” Hepatology Research 54, no. 8 (2024): 763–772. 38638067 10.1111/hepr.14047

[5] M. E. Rinella , J. V. Lazarus , V. Ratziu , et al., “A Multisociety Delphi Consensus Statement on New Fatty Liver Disease Nomenclature,” Hepatology 78, no. 6 (2023): 1966–1986. 37363821 10.1097/HEP.0000000000000520 PMC10653297

[6] K. Suzuki , N. Tamaki , M. Kurosaki , et al., “Concordance Between Metabolic Dysfunction‐Associated Steatotic Liver Disease and Nonalcoholic Fatty Liver Disease,” Hepatology Research 54, no. 6 (2024): 600–605. 38234088 10.1111/hepr.14011

[7] K. Tokushige , “New Concept in Fatty Liver Diseases,” Hepatology Research 54, no. 2 (2024): 125–130. 38146790 10.1111/hepr.14004

[8] D. Q. Huang , N. A. Terrault , F. Tacke , et al., “Global Epidemiology of Cirrhosis—Aetiology, Trends and Predictions,” Nature Reviews. Gastroenterology and Hepatology 20, no. 6 (2023): 388–398. 36977794 10.1038/s41575-023-00759-2 PMC10043867

[9] T. Kimura , N. Tamaki , S. I. Wakabayashi , et al., “Colorectal Cancer Incidence in Steatotic Liver Disease (MASLD, MetALD, and ALD),” Clinical Gastroenterology and Hepatology (2025). 10.1016/j.cgh.2024.12.018 PMC12441595 39892626

[10] N. Tamaki , M. Higuchi , M. Kurosaki , R. Loomba , and N. Izumi , “Risk Difference of Liver‐Related and Cardiovascular Events by Liver Fibrosis Status in Nonalcoholic Fatty Liver Disease,” Clinical Gastroenterology and Hepatology 20, no. 5 (2022): 1171–1173.e2. 34280550 10.1016/j.cgh.2021.07.021 PMC8761224

[11] N. Tamaki , T. Kimura , S. I. Wakabayashi , et al., “Long‐Term Clinical Outcomes in Steatotic Liver Disease and Incidence of Liver‐Related Events, Cardiovascular Events and All‐Cause Mortality,” Alimentary Pharmacology and Therapeutics 60, no. 1 (2024): 61–69. 38664876 10.1111/apt.18015

[12] S. I. Wakabayashi , N. Tamaki , T. Kimura , T. Umemura , M. Kurosaki , and N. Izumi , “Natural History of Lean and Non‐Lean Metabolic Dysfunction‐Associated Steatotic Liver Disease,” Journal of Gastroenterology 59, no. 6 (2024): 494–503. 38570344 10.1007/s00535-024-02093-z

[13] S. Yamashita , D. Masuda , and Y. Matsuzawa , “Pemafibrate, a New Selective PPARα Modulator: Drug Concept and Its Clinical Applications for Dyslipidemia and Metabolic Diseases,” Current Atherosclerosis Reports 22, no. 1 (2020): 5. 31974794 10.1007/s11883-020-0823-5 PMC6978439

[14] K. Tsuji , N. Tamaki , M. Kurosaki , et al., “Pemafibrate Improves Liver Biochemistry and GLOBE Scores in Patients With Primary Biliary Cholangitis: Nationwide, Multicenter Study by the Japanese Red Cross Liver Study Group,” Hepatology Research 55, no. 5 (2025): 675–684. 40317593 10.1111/hepr.14172

[15] T. Hatanaka , S. Kakizaki , N. Saito , et al., “Impact of Pemafibrate in Patients With Hypertriglyceridemia and Metabolic Dysfunction‐Associated Fatty Liver Disease Pathologically Diagnosed With Non‐Alcoholic Steatohepatitis: A Retrospective, Single‐Arm Study,” Internal Medicine 60, no. 14 (2021): 2167–2174. 33612679 10.2169/internalmedicine.6574-20 PMC8355409

[16] A. Nakajima , Y. Eguchi , M. Yoneda , et al., “Randomised Clinical Trial: Pemafibrate, a Novel Selective Peroxisome Proliferator‐Activated Receptor α Modulator (SPPARMα), Versus Placebo in Patients With Non‐Alcoholic Fatty Liver Disease,” Alimentary Pharmacology and Therapeutics 54, no. 10 (2021): 1263–1277. 34528723 10.1111/apt.16596 PMC9292296

[17] T. Iwadare , T. Kimura , H. Kunimoto , et al., “Higher Responsiveness for Women, High Transaminase Levels, and Fat Percentage to Pemafibrate Treatment for NAFLD,” Biomedicine 10, no. 11 (2022): 2806. 10.3390/biomedicines10112806 PMC9687993 36359326

[18] A. Morishita , K. Oura , K. Takuma , et al., “Pemafibrate Improves Liver Dysfunction and Non‐Invasive Surrogates for Liver Fibrosis in Patients With Non‐Alcoholic Fatty Liver Disease With Hypertriglyceridemia: A Multicenter Study,” Hepatology International 17, no. 3 (2023): 606–614. 36583842 10.1007/s12072-022-10453-1 PMC10224826

[19] R. Sugimoto , M. Iwasa , A. Eguchi , et al., “Effect of Pemafibrate on Liver Enzymes and Shear Wave Velocity in Non‐Alcoholic Fatty Liver Disease Patients,” Frontiers in Medicine 10 (2023): 1073025. 36824614 10.3389/fmed.2023.1073025 PMC9941328

[20] Y. Suzuki , S. Maekawa , K. Yamashita , et al., “Effect of a Combination of Pemafibrate and a Mild Low‐Carbohydrate Diet on Obese and Non‐Obese Patients With Metabolic‐Associated Fatty Liver Disease,” Journal of Gastroenterology and Hepatology 38, no. 6 (2023): 921–929. 36811251 10.1111/jgh.16154

[21] Y. Takahashi , Y. Seko , K. Yamaguchi , et al., “Gamma‐Glutamyl Transferase Predicts Pemafibrate Treatment Response in Non‐Alcoholic Fatty Liver Disease,” Journal of Gastroenterology and Hepatology 38, no. 10 (2023): 1743–1749. 37221601 10.1111/jgh.16222

[22] M. Yamada‐Shimizu , N. Tamaki , M. Kurosaki , et al., “A Comparison of Alanine Aminotransferase Normalization Between Pemafibrate and Bezafibrate in Patients With Nonalcoholic Fatty Liver Disease,” Internal Medicine 63, no. 9 (2024): 1185–1190. 37779070 10.2169/internalmedicine.2248-23 PMC11116030

[23] S. Shinozaki , K. Miura , T. Tahara , and H. Yamamoto , “Effectiveness of Pemafibrate Dose Escalation on Metabolic Dysfunction‐Associated Steatotic Liver Disease Refractory to Standard Dose,” Metabolites 15, no. 2 (2025): 100. 39997725 10.3390/metabo15020100 PMC11857616

[24] Y. Sumida , H. Toyoda , S. Yasuda , et al., “Comparison of Efficacy Between Pemafibrate and Omega‐3‐Acid Ethyl Ester in the Liver: The PORTRAIT Study,” Journal of Atherosclerosis and Thrombosis 31, no. 11 (2024): 1620–1633. 38777770 10.5551/jat.64896 PMC11537790

[25] M. Iwasa , R. Sugimoto , A. Eguchi , et al., “Effectiveness of 1‐Year Pemafibrate Treatment on Steatotic Liver Disease: The Influence of Alcohol Consumption,” European Journal of Gastroenterology and Hepatology 36, no. 6 (2024): 793–801. 38526942 10.1097/MEG.0000000000002766

[26] Y. Takeda , M. Furuhashi , I. Sakuma , et al., “Comparison of the Effects of Pemafibrate and Omega‐3 Fatty Acid Ethyl on Fatty Liver Index in Patients With Dyslipidemia Treated With Statin: A Sub‐Analysis From the PROUD48 Study,” Frontiers in Endocrinology 16 (2025): 1549687. 40375947 10.3389/fendo.2025.1549687 PMC12078148

[27] T. Ichikawa , M. Yamashima , S. Yamamichi , et al., “Pemafibrate Reduced Liver Stiffness in Patients With Metabolic Dysfunction‐Associated Steatotic Liver Disease Complicated With Hyperlipidemia and Liver Fibrosis With a Fibrosis‐4 Index Above 1.3,” Internal Medicine 64, no. 9 (2025): 1296–1302. 39293976 10.2169/internalmedicine.4337-24 PMC12120207

[28] Y. Kanda , “Investigation of the Freely Available Easy‐To‐Use Software EZR for Medical Statistics,” Bone Marrow Transplantation 48, no. 3 (2013): 452–458. 23208313 10.1038/bmt.2012.244 PMC3590441

[29] K. Tokushige , K. Ikejima , M. Ono , et al., “Evidence‐Based Clinical Practice Guidelines for Nonalcoholic Fatty Liver Disease/Nonalcoholic Steatohepatitis 2020,” Journal of Gastroenterology 56, no. 11 (2021): 951–963. 34533632 10.1007/s00535-021-01796-x PMC8531062

[30] K. Tokushige , K. Ikejima , M. Ono , et al., “Evidence‐Based Clinical Practice Guidelines for Nonalcoholic Fatty Liver Disease/Nonalcoholic Steatohepatitis 2020,” Hepatology Research 51, no. 10 (2021): 1013–1025. 34533266 10.1111/hepr.13688

[31] “EASL‐EASD‐EASO Clinical Practice Guidelines on the Management of Metabolic Dysfunction‐Associated Steatotic Liver Disease (MASLD),” Journal of Hepatology 81, no. 3 (2024): 492–542. 38851997 10.1016/j.jhep.2024.04.031

[32] F. Kanwal , B. A. Neuschwander‐Tetri , R. Loomba , and M. E. Rinella , “Metabolic Dysfunction‐Associated Steatotic Liver Disease: Update and Impact of New Nomenclature on the American Association for the Study of Liver Diseases Practice Guidance on Nonalcoholic Fatty Liver Disease,” Hepatology 79, no. 5 (2024): 1212–1219. 38445559 10.1097/HEP.0000000000000670 PMC13435502

[33] D. Q. Huang , S. R. Sharpton , M. Amangurbanova , N. Tamaki , C. B. Sirlin , and R. Loomba , “Clinical Utility of Combined MRI‐PDFF and ALT Response in Predicting Histologic Response in Nonalcoholic Fatty Liver Disease,” Clinical Gastroenterology and Hepatology 21, no. 10 (2023): 2682–2685.e4. 36075503 10.1016/j.cgh.2022.08.036

[34] N. Akuta , Y. Kawamura , S. Fujiyama , et al., “Treatment Efficacy of Diet and Exercise Program for Fatty Liver and Pretreatment Predictors,” Hepatology Research 53, no. 7 (2023): 607–617. 36891614 10.1111/hepr.13897

[35] Y. Komiyama , U. Motosugi , S. Maekawa , et al., “Early Diagnosis of Hepatic Inflammation in Japanese Nonalcoholic Fatty Liver Disease Patients Using 3D MR Elastography,” Hepatology Research 53, no. 3 (2023): 208–218. 36372908 10.1111/hepr.13858 PMC10600503

